# Accidental Ante-Partum Hemorrhage

**Published:** 1882-01

**Authors:** 


### SELECTIONS.

### ACCIDENTAL ANTE-PARTUM HEMORRHAGE.

Dr. Edward L. Partridge, of New York, Physician to the Nursery and Child's Hospital, contributes to the December number of the *New York Medical Journal and Obstetrical Review* an article in which, after briefly reviewing the current doctrines concerning so-called accidental hemorrhage preceding the birth of the child, he boldly challenges the expediency of the practice of rupturing the membranes. He believes, first, that rupture of the membranes does not meet the indications—i. e., it does not in itself or in its results offer any reasonable probability of checking the hemorrhage—and, secondly, that the method is highly dangerous from the increase of facilities for loss of blood, and because it adds to the difficulty and danger of the proper subsequent steps in treatment. As to whether it really does check hemorrhage, it cannot do so unless a decided decrease in uterine bulk can be secured and maintained thereby. There must, therefore, be a considerable number of cases in which, a small amount of liquor amnii being present and the reduction in size being very slight after its escape, no benefit can accrue. In cases which present an average amount of amniotic fluid, after its evacuation the uterus is decidedly, though not greatly, diminished in size. What is to show, however, that this decrease is sufficient to close the mouths of bleeding vessels? There is no practitioner who cannot affirm that alarming hemorrhage does often threaten after the birth of the child, and before or after the complete separation of the placenta, when the uterus is *greatly contracted.* Even this degree in the reduction of bulk fails to close the uterine sinuses in the intervals of contraction. All those writers who advise rupture of the membranes couple with this advice the information that there is danger of continued hemorrhage. One says, "Of course, there is risk," while all suggest methods by which they think a loss and a large accumulation of blood can be prevented after the escape of the amniotic fluid—these suggestions looking toward the maintenance of contraction. Accidental hemorrhage usually takes place prior to or during the occurrence of infrequent and slight early uterine contractions, when the os is slightly dilated or not at all. Superadded is the condition of collapse. If the liquor amnii is now permitted to escape, can any candid, practical obstetrician admit, the author asks, that there is any known way by which a momentary reduction of uterine bulk can be maintained for a period which will check an alarming hemorrhage? The uncertainties and tediousness of efforts at excitation of the uterus in cases of induction of labor afford a good illustration of the difficulties which would be encountered. Ergot is uncertain and almost valueless, for the stomach will either reject or fail to absorb it; or, if absorption does take place, or if the drug is given by the hypodermic method, its action is imperfect when there has been a great drain upon the vital powers. The abdominal binder cannot be applied in a way to crowd the resilient uterine tissue into contraction. Manual efforts cannot be kept up with any precision or efficacy during the period necessary to check the hemorrhage and keep it in control. Good uterine action cannot be excited when the uterus is surprised into labor. Good labor-pains will not occur when the patient is exsanguinated. The suggestion of Leishman, to the effect that the placenta will be compressed between the uterus and the child after the escape of the liquor amnii, and hemorrhage thus be checked, is, Dr. Partridge thinks, fanciful; for no sufficient uterine action will take place to effect this. There are a great many chances also that the part of the child nearest the placenta would not be one which could make an even, perfect compression, if suitable uterine action did take place. Far from meeting the emergency, the method greatly increases the dangers. If the uterus does not contract promptly and permanently after the escape of the liquor amnii, an ample space is afforded for a further extravasation of blood. A very limited space will afford room for a dangerous extravasation. Another danger is from a more extensive detachment of the placenta when the uterus is even temporarily contracted. Another objection to the early removal of the liquor amnii in accidental hemorrhage is, that an obstacle is created to the use of the most efficient method for securing dilatation of the os—i. e., by the dilators. Their use would be improper, lest, acting also as a tampon, they should prevent egress of effused blood, and add to to the accumulation. A fourth danger will be from the increased difficulty encountered in the performance of version if the child is not surrounded by liquor amnii. This operation is often imperatively demanded in the treatment of accidental hemorrhage, under circumstances, too, when its ease of performance is of great importance. There is one class of cases of accidental hemorrhage in which the amount of blood lost does not fully explain the degree of shock. In these the factors in the production of collapse are the over-distension of the uterus and consequent irritation of the peripheral nerves of that organ, as well as the abstraction of blood from the circulation. Here, then, we might believe, was found sufficient ground for the treatment by early rupture of the membranes, relieving thereby uterine distention and the resulting irritation to the nervous system. Upon consideration, however, we find, first, that it is impossible to prejudge in these cases. It is only *after* delivery, when the amount of effused blood can be estimated, that we discover that the shock was proportionately greater than the hemorrhage. Again, collapse brought about in this way does not obstinately refuse to yield to treatment, but will be remedied usually by the customary measures, such as stimulants, the application of external heat, etc., without the need of any decided local interference. Finally, this variety of the accident is not very common, as indicated by clinical records, the possibility of its occurrence being so lightly regarded as hardly to be mentioned by writers. What, then, should be the treatment looking toward the safety of mother and child when immediate delivery cannot be resorted to, owing to incomplete dilatation of the os? By all means *preserve the membranes intact* and thus tampon the uterine cavity with liquor amnii. Then, in the great majority cases, employ Barnes' dilators until the desired result is obtained. Of course, this or any similar treatment must be employed at a suitable time. It must not supersede efforts for the relief of collapse, and it may be necessary to defer all operative measures until the patient can be rallied from the alarming constitutional symptoms. The os being sufficiently dilated to enable delivery to take place, rupture of the membranes is proper, and should be followed by manual efforts to compel the uterus to descend upon the child, whose expulsion should be immediate. Version fulfils the indications better than the forceps, as by the former operation there is less danger from delay during delivery, and because it can be successfully resorted to at an earlier period in the dilatation of the os than the forceps can. Bimanual version should not be considered for a moment, as in cases apparently most favorable it cannot always be accomplished, while in this accident the irregularity of the internal uterine surface caused by the collection of blood would certainly interfere with the change of position of the child. During the entire time stimulants must be freely used and warmth to the surface, and in exceptional cases, when the hemorrhage does not appear to be continuing, it is proper to wait for returning vitality before operative measures are undertaken, lest the condition of collapse be aggravated. The danger is not necessarily over after delivery, for it is often difficult to bring about reaction from the dangerous condition, and convalescence will often be slow.

